# Clinical evolution of Catatonia, the role of Bush Francis Catatonia Rating Scale in case series

**DOI:** 10.1192/j.eurpsy.2026.11690

**Published:** 2026-07-31

**Authors:** E. C. Ezema, E. U. Ezenagu, S. Singh, A. Meftah, J. Beauchamp, S. Argyriou, T. Olupona

**Affiliations:** Psychiatry, One Brooklyn Health-Interfaith , Brooklyn, United States

## Abstract

**Introduction:**

Catatonia is a complex neuropsychiatric behavior typified by negativism, mutism, stupor, excitement, stereotyped movement, staring, grimacing, echolalia, and echopraxia. At least, 3 symptoms are required for diagnosis. Immediate intervention is crucial to prevent complications which includes dehydrations, malnutrition, deep venous thrombosis, decubitus ulcer, acute renal failure, and ocular drying. Therefore, the use of a screening tool like Bush Francis Catatonia Rating Scale (BFCRS) in timely interval is imperative for a successful monitoring of the clinical progress. We present 3 cases of catatonia, their clinical trajectories were aided by the BFCRS.

**Objectives:**

To evaluate the impact of BFCRS on the diagnosis and recovery of catatonia

**Methods:**

Case 1: A 61-year-old African-American female with a diagnosis of schizophrenia, presented with disorganized behavior and admitted as a case of schizophrenia, with acute exacerbation. On evaluation, muteness, stupor, and fixed staring were noted. The initial BFCRS score was 10, supporting the diagnosis of schizophrenic catatonia. The patient was started on Lorazepam 2 mg tid (IM, on declining oral) and Risperidone 1 mg PO bid. The clinical progress was monitored with BFCRS and the score gradually decreased to 0 when the patient was clinically stable, discharged on Risperidone 2 mg PO bid.

Case 2: A 31-year-old African-American female with a diagnosis of Cerebral palsy and Schizophrenia, presented initially with toxic metabolic encephalopathy following poor oral intake of food and fluids for 3 days. The patient exhibited mutism, stupor, echolalia, staring, grimacing, ambitendency and verbigeration with a fluctuating course. A diagnosis of schizophrenic-catatonia was made. The patient was started on Lorazepam 2 mg tid (IM, on declining oral). Her clinical progress was monitored with BFCRS and gradually decreased from 9 to 0 as the patient improved. The patient was stabilized and discharged on Olanzapine 15 mg PO daily.

Case 3: A case of a 34-year-old African-American Male with a diagnosis of schizoaffective disorder, depression, cocaine, and cannabis use disorder, presented on account of suicidal ideation and not eating. The patient was verbally unresponsive, sitting still, and staring. He was diagnosed with depressive-catatonia and started on Lorazepam 2 mg tid (IM, on declining oral), He was also started on Wellbutrin 150 mg XL daily. Monitoring of clinical improvement was aided by BFCRS which was initially 8 and gradually reduced to 0 when catatonic features were resolved.

**Results:**

The employment of BFCRS played a role in monitoring the evolution of the patients’ condition until resolution. BFCRS not only helped in supporting the diagnoses of the cases but was a resourceful tool in the care plan of the patients.

**Conclusions:**

The application of BFCRS is crucial, not only aiding diagnosis of catatonia but also assist in monitoring the clinical trajectory

**Disclosure of Interest:**

None Declared

